# Achilles tendon thickness reduces immediately after a marathon

**DOI:** 10.1186/s13018-022-03448-z

**Published:** 2022-12-23

**Authors:** Isabelle Scott, Peter Malliaras, Alex Tardioli, Sarah Hales, Dylan Morrissey, Filippo Migliorini, Nicola Maffulli

**Affiliations:** 1 Department of Mathematics, University of Oxford, Oxford, England; 2 Centre for Sports and Exercise Medicine, Barts and The London School of Medicine and Dentistry, Mile End Hospital, Queen Mary University of London, London, E1 4DG England; 3 Department of Physiotherapy, Monash University, Melbourne, VIC Australia; 4 Department of Orthopaedic, Trauma, and Reconstructive Surgery, RWTH University Hospital, Pauwelsstraße 30, 52074 Aachen, Germany; 5 Department of Medicine, Surgery and Dentistry, University of Salerno, 84081 Baronissi, SA Italy; 6 Faculty of Medicine, School of Pharmacy and Bioengineering, Keele University, Stoke on Trent, ST4 7QB England

**Keywords:** Achilles tendon, Fluid flux, Marathon, Tendinopathy, Ultrasound

## Abstract

**Background:**

The purpose of the present investigation was to evaluate the immediate effect of running a marathon on Achilles tendon anteroposterior thickness.

**Methods:**

In 25 runners who took part in the London marathon, ultrasonography was used to measure the Achilles tendon thickness pre- and immediately post-marathon and to identify any structural abnormalities indicating tendinopathy. Pain was recorded using a numerical rating scale at baseline and post-marathon. Twenty-one participants were included in the final analysis.

**Results:**

Running a marathon resulted in a significant decrease (− 13%, *p* < 0.01) in anteroposterior diameter of the Achilles tendon immediately following the marathon. There was no change in the proportion of Achilles tendons with structural abnormalities (34%) or pain (12%) following the marathon (*p* > 0.05).

**Conclusion:**

Running a marathon resulted in an immediate reduction in anteroposterior diameter of the Achilles tendon. This finding may have implications for injury prevention and recovery following a marathon.

## Background

Tendinopathy is a common musculoskeletal condition producing significant morbidity. Achilles tendinopathy is common, with a particularly high prevalence in runners [1]. Proteins in the collagen and ground substance matrix are produced by tendon cells (tenocytes) that are able to detect and respond to mechanical load [2]. Chronic tendinopathy is associated with accumulation of ground substance, intratendinous collagen disarray, and disordered neoangiogenesis [1, 3]. Excessive repeated loading is an important aetiological factor in Achilles tendinopathy. However, carefully progressed loading is also an essential stimulus for positive symptomatic and adaptive changes [4]. The mechanisms by which these changes occur are still not fully understood. Insight into the effects of different forms of mechanical loading on tendons should allow better understand both injurious and rehabilitative mechanisms.

Several studies have reported a reduction in Achilles tendon thickness following acute bouts of loading, including eccentric ankle loading exercises [5, 6] and a match of floor-ball [7]. Given the timeframe needed for collagen remodelling to occur, immediate decrease in thickness likely indicates fluid loss. Previous in vitro and computational work has shown that intratendinous fluid content significantly influences stress relaxation and stiffness within the tendon [8–10]. Hence, loading of the tendon whilst it is fluid depleted may play a role in the pathoaetiology of tendinopathy. Changes in thickness could also result from changes in the resting tendon length [11].

Whilst no significant reduction in anteroposterior diameter was detected 40 h after a marathon [12], the immediate effects of running a marathon on Achilles tendon thickness are poorly studied. This is important, as alterations in thickness and hydration are usually detectable only within the first 24 h of the exercise bout [13, 14] and changes in thickness following a marathon may expose the Achilles tendon to injury. The primary aim of this study was to determine whether Achilles anteroposterior diameter changes immediately after a marathon.

## Materials and methods

### Participants

Twenty-five experienced runners (all had run more than 20 km per week (range 20–45), on average, for the last 5 years) from running clubs in the local London area were recruited to participate in the study. Runners were included if they planned to complete the full London marathon and had at least one Achilles tendon without a history of rupture or surgery. Participants were non-smokers and were not taking medication known to affect tendon structure (e.g. fluoroquinolones and statins). Runners provided written informed consent, and ethical approval was obtained from the Queen Mary University Research and Ethics Committee [15].

### Procedure

Participants presented for baseline imaging at the marathon registration centre. They completed a questionnaire collecting demographic (age and gender) and activity (weekly running volume and estimated marathon time) data. Current Achilles tendon pain status was determined by the presence of exercise-related localised Achilles pain and morning stiffness. This was performed by another researcher (AT) so that the ultrasonographer was blind to clinical status. Participants were seated for a minimum of 30 min and had not engaged in sporting activity 24 h prior to baseline ultrasound assessments. Follow-up data were collected within five to 15 min of marathon completion, including current pain status and ultrasound imaging.

### Ultrasound

**Fig. 1:**
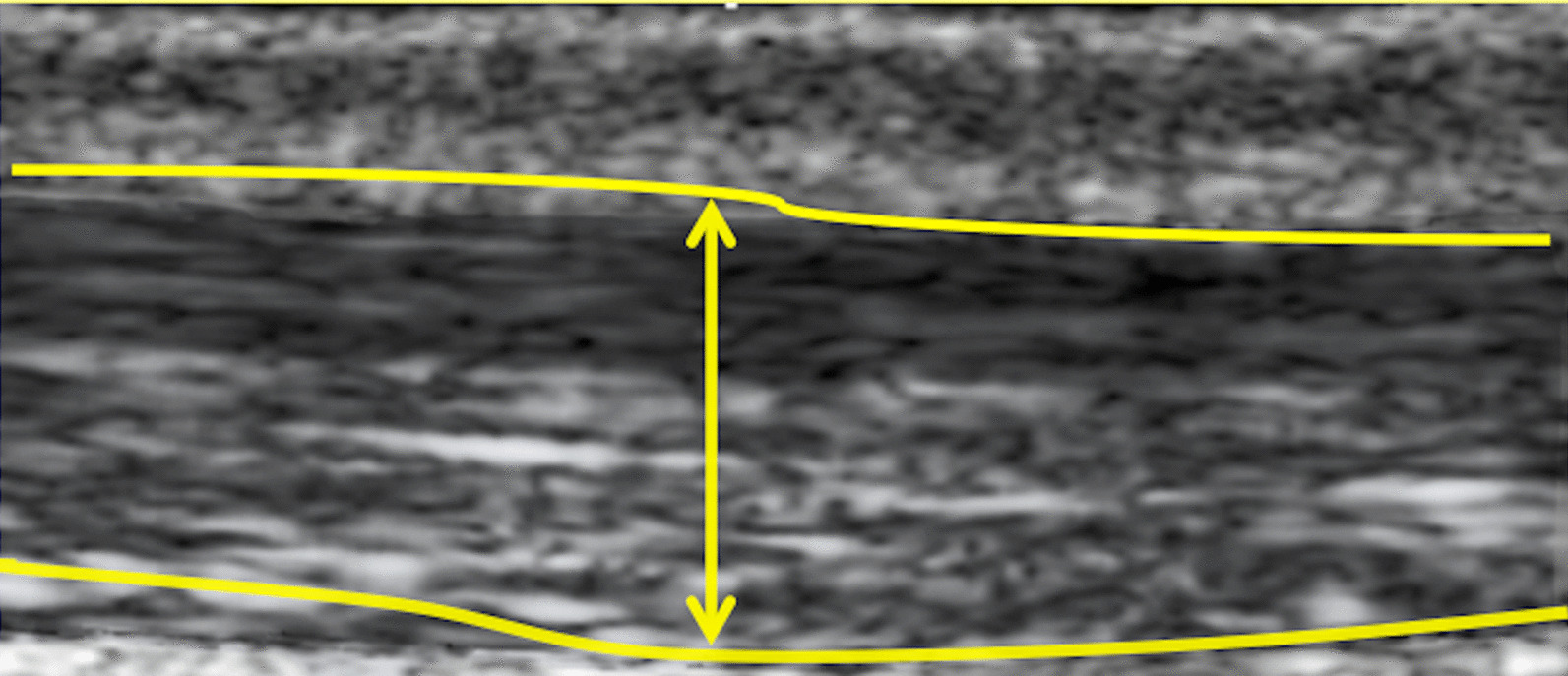
Example of measurement of the anteroposterior (AP) thickness of an Achilles tendon

Tendons were examined using real-time grey-scale B-mode ultrasound and power Doppler with a high-resolution, portable ultrasound system (Voluson I, GE Healthcare, London, UK) equipped with a 3–12 MHz linear transducer. Power Doppler frequency was set to 7 MHz, and the gain was set just below the level that produced random noise. Ultrasound examinations were performed by a radiology trainee (SH) with experience of imaging over 200 Achilles tendons. Participants lay prone with their feet hanging over the end of the medical plinth and pointing directly downward. Tendons were imaged in the sagittal and axial planes, taking care to avoid anisotropy. The anteroposterior diameter of the Achilles tendon was measured from axial ultrasound images [16] at the midportion of the Achilles tendon (Fig. 1) [5, 6, 11]. Echogenicity of the Achilles tendon was rated as normal or hypoechoic, and power Doppler signal was assessed (present or absent) [17].

### Statistical evaluation

Statistical analysis was performed using SPSS software (version 20.0; SPSS Inc., Chicago, Illinois). Nonparametric tests were used with the level of significance set at 0.05. The change in anteroposterior diameter of the Achilles tendon before and after the marathon was assessed by the Wilcoxon’s matched pairs signed rank test. The Mann–Whitney *U* test was used to further investigate group differences. The changes in anteroposterior diameter were compared between ecographically normal and abnormal Achilles tendons (defined as the presence of a hypoechoic region) on baseline ultrasound images.

## Results

Twenty-one runners (15 men and 6 women) were included in the final analysis, as four failed to attend for post-marathon imaging. Forty-one Achilles tendons were included in the study (1 excluded because of previous rupture). Baseline descriptive data and anteroposterior diameter are shown in Table 1. Ten tendons were abnormal on ultrasound (hypoechoic region with or without Doppler signal) and five participants had Achilles tendon pain. There was no change in pain status, or the presence of ultrasound abnormalities from baseline to immediately post-marathon.

**Table 1:** Descriptive data for the entire cohort

Endpoints	Median (IQR)
Age	40 (13)
Running volume (km/week)	58 (20)
Marathon time (hr:min)	03:45 (00:33)
Anteroposterior thickness (mm)	5.3 (1.0)

**Fig. 2:**
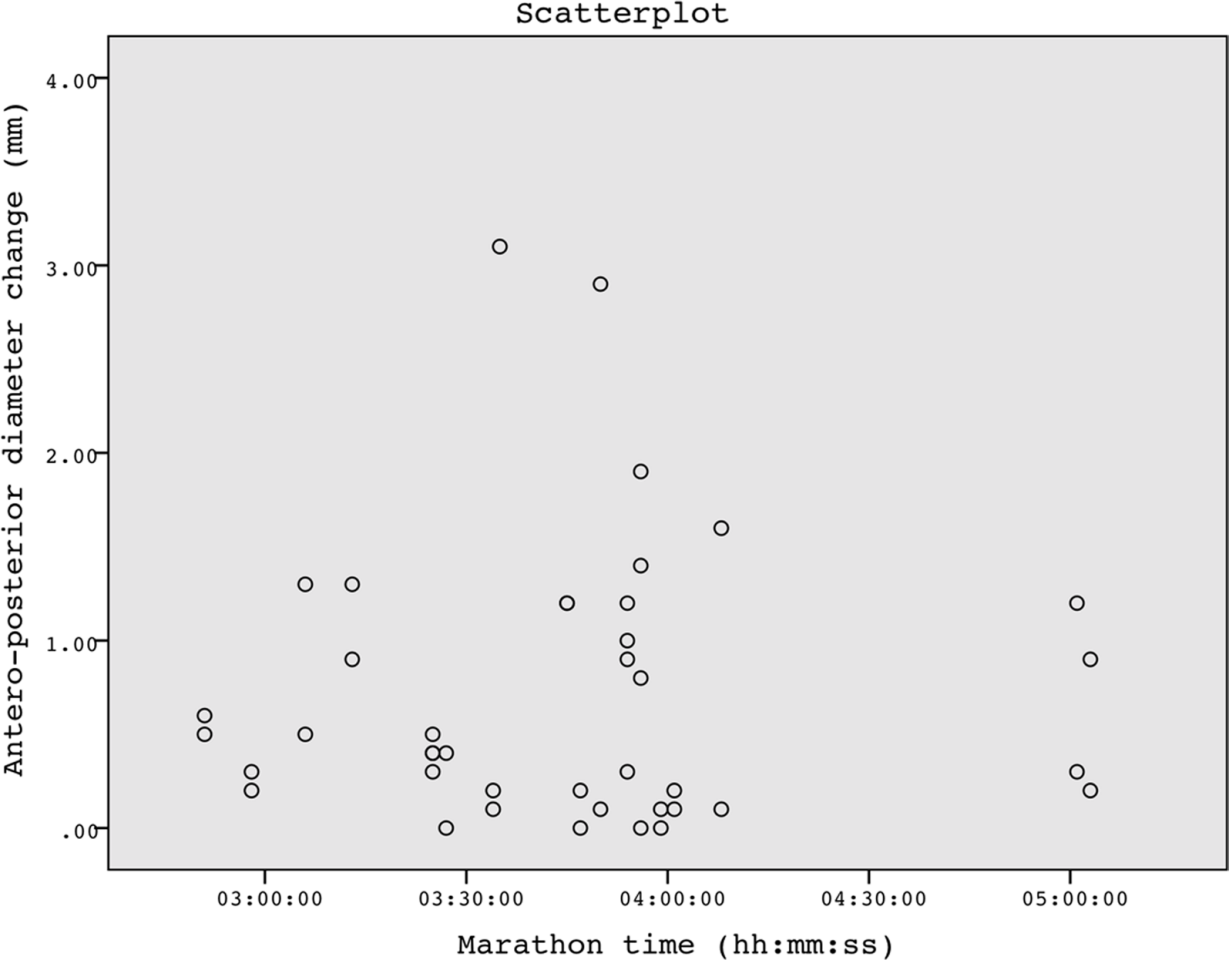
Changes in anteroposterior diameter of the Achilles tendon before and immediately after the marathon according to the time to its completion

The anteroposterior diameter decreased significantly (mean 0.7 mm, − 13%) from baseline to immediately post-marathon (Wilcoxon matched pairs signed-rank test, *p* < 0.01, Fig. 2).

**Fig. 3:**
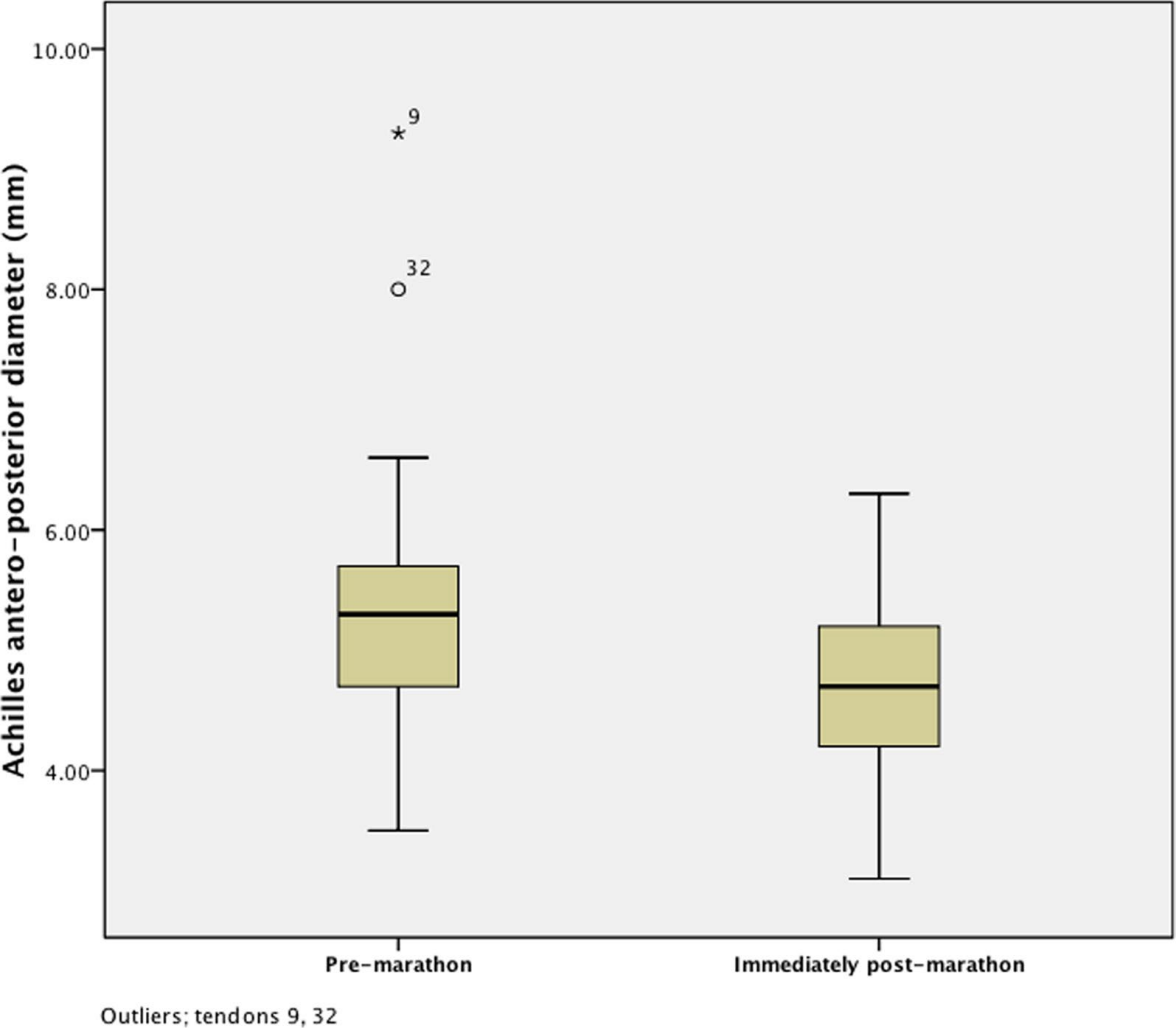
Anteroposterior diameter of the tendons before and immediately after having completed the marathon

The median anteroposterior diameter decreased from 5.3 mm (IQR 0.7 mm) pre-marathon to 5 mm (IQR 1 mm) immediately post-marathon (Fig. 3). The changes in anteroposterior diameter were not significantly different between abnormal and normal tendons (Mann–Whitney *U*, *p* = 0.20).

## Discussion

This study investigated the immediate changes in Achilles tendon anteroposterior diameter following a marathon, showing a significant reduction in its anteroposterior diameter. Ooi et al. [12] found no significant reduction in anteroposterior diameter 40 h after a marathon. The present work would indicate immediate transient Achilles tendon thickness changes in response to prolonged running.

This finding may enable us to better understand the biomechanical changes which occur with loading, helping identify the potential mechanisms of tendinopathy. There are, however, several limitations to the present study. For example, the anteroposterior diameter of the Achilles tendon was assessed at one site and at one time point post-marathon. Adding cross-sectional area and anteroposterior diameter at other sites would have provided a more complete picture of tendon volume changes and potential fluid flux, while adding measurements at several different time intervals post-marathon would have given insight into the recovery time of these changes. Additionally, measurement of an upper limb tendon would have allowed us to ascertain whether fluid losses were limited to the lower limb or were more generalised. We acknowledge that there was no control group, but tendon thickness is unlikely to change without an exercise bout [5]. Also, the radiologist was not blind to the activity undertaken by the athletes examined. Potential confounders such as relative performance to previous marathon times or whether a subject was a forefoot or rear foot striker were not assessed. Furthermore, pain severity could have been recorded with a validated condition-specific outcome.

The reduction in anteroposterior diameter likely represents fluid loss from the tendon. Fluid loss with loading has been demonstrated in multiple in vitro [18] and computational [9, 19] studies, as well as standard MR off-resonance saturation pulse imaging work which demonstrated a reduction in tendon volume and hydration status following both a 3.9 and 6.6 km run [14]. Similar to the reduction in anteroposterior diameter observed in the present investigation (− 0.7 mm, − 13%), significant reductions were found after six sets (3 straight and 3 bent leg) of 15 eccentric ankle exercises (− 0.9 mm, − 20%) [6] and a one-hour floor-ball match (− 0.3 mm, − 5%) [7].

In contrast to the reduction in tendon anteroposterior diameter detected after a marathon, no change in the average Achilles tendon cross-sectional area (CSA) was detected after five kilometres [20] or 30-min runs [21]. Both Lichtwark et al. [20] and Farris et al. [21] measured CSA at multiple points along the tendon, so it is likely that they employed a more sensitive methodology to detect changes in tendon size. This suggests that shorter running interventions may not be sufficient to result in a change in tendon size. An alternative hypothesis is that fluid is redistributed, and therefore, the CSA is unchanged, even though the anteroposterior diameter may have changed at certain points along the tendon. In support of this, Neves et al. [22] reported a reduction in the CSA (measured at a single point along the tendon) of the Achilles tendon on ultrasound imaging after 10 min of treadmill running. Further, an increase in tendon resting length may explain the reduction, having been shown to occur following both eccentric ankle exercises and running [11, 20]. More work is needed to understand whether the post-marathon changes in anteroposterior diameter we observed relate to fluid flow that is sensitive to running volume, tendon length changes, fluid redistribution or other mechanisms. In vitro and computational studies have demonstrated that greater interstitial fluid corresponds to enhanced stress relaxation and stiffness [8, 9], especially with high-strain-rate loading [10]. It has been suggested that fluid loss exposes the tendon to greater loads [23], possibly explaining why inadequate recovery time has been identified as a risk factor for tendinopathy [3]. It may be of benefit to evaluate tendon thickness following key sporting engagements to determine optimal recovery time. Further work should evaluate the likely timeframes of fluid recovery. This will provide guidance for clinicians in determining the optimum frequency of loading for prevention and rehabilitation of tendinopathy.

Tendinopathic tendons exhibit an increase in ground substance with associated tendon thickening. Fluid loss has the potential to affect tenocytes through fluid-induced shear stress, and the lack of surrounding fluid may limit protection from compression by surrounding solid components. In response to compressive overload, tenocytes produce hydrophilic molecules which bind water [23]. Tendon thickening and the blunted anteroposterior diameter response to load seen in tendinopathy [5] may be an attempt to protect against higher running loads associated with fluid loss. We did not observe this phenomenon in the present study probably given the small number of pathological tendons. This would explain the association between increased midportion diameter and tendinopathy [24]. Future studies should attempt to elucidate the relationship between changes in 3D tendon volume, anteroposterior diameter, and fluid loss. They should aim to determine whether reductions in anteroposterior diameter represent fluid redistribution or fluid loss from the tendon, how long the changes take to recover, and how this relates to the strains imposed on the Achilles tendon and injury risk. Further insight into fluid changes with loading may give key insight into the mechanisms of tendinopathy and help guide clinicians in preventing overload of the tendon from occurring. Finally, the lack of validated patients’ reported outcome measures (PROMs), such as the Victorian Institute of Sport Assessment- Achilles (VISA-A) questionnaire and additional patients characteristics (e.g. BMI), represents an important limitation of the present study, which should be implemented in future investigations.

## Data Availability

The datasets generated during and/or analysed during the current study are available throughout the manuscript.

## Abbreviation

CSA: Cross-sectional area
